# SeV C Protein Plays a Role in Restricting Macrophage Phagocytosis by Limiting the Generation of Intracellular Double-Stranded RNA

**DOI:** 10.3389/fmicb.2022.780534

**Published:** 2022-02-21

**Authors:** Naoko Morita, Yukie Tanaka, Kenji Takeuchi, Yoshinori Kitagawa, Ryusuke Sakuma, Naoki Koide, Takayuki Komatsu

**Affiliations:** ^1^Department of Microbiology and Immunology, Aichi Medical University School of Medicine, Aichi, Japan; ^2^Department of Integrative Vascular Biology, Faculty of Medical Sciences, University of Fukui, Fukui, Japan; ^3^Department of Genome Science and Microbiology, Faculty of Medical Sciences, University of Fukui, Fukui, Japan; ^4^Division of Microbiology and Infectious Diseases, Department of Pathology, Shiga University of Medical Science, Shiga, Japan

**Keywords:** Sendai virus, C protein, double-stranded RNA, RIG-I, macrophage, phagocytosis

## Abstract

Macrophages play a central role in the innate immune response to respiratory viral infections through pro-inflammatory factor secretion and phagocytosis. However, as a countermeasure, viral pathogens have evolved virulence factors to antagonize macrophage function. In our recent *in vitro* analyses of murine macrophage cell lines, Sendai virus (SeV) accessory protein C inhibited the secretion of pro-inflammatory factors, and C gene-knockout SeV (SeVΔC) caused drastic morphological changes in RAW264.7 macrophages, similar to those observed after stimulation with Lipid A, a well-known activator of actin-rich membrane ruffle formation and phagocytosis. Hence, we sought to determine whether the C protein limits phagocytosis in SeV-infected macrophages through the suppression of membrane ruffling. Phagocytosis assays indicated an upregulation of phagocytosis in both SeVΔC-infected and Lipid A-stimulated macrophages, but not in SeV WT-infected cells. Further, the observed membrane ruffling was associated with phagocytosis. RIG-I is essential for Lipid A-induced phagocytosis; its deficiency inhibited SeVΔC-stimulated phagocytosis and ruffling, confirming the essential role of RIG-I. Moreover, treatment with interferon (IFN)-β stimulation and neutralizing antibodies against IFN-β suggested that SeVΔC-induced phagocytosis and ruffling occurred in an IFN-β-independent manner. A newly isolated SeVΔC strain that does not generate dsRNA further highlighted the importance of dsRNA in the induction of phagocytosis and ruffling. Taken together, the current results suggest that SeV C protein might limit phagocytosis-associated membrane ruffling in an RIG-I-mediated but IFN-independent manner via limiting the generation of intracellular dsRNA.

## Introduction

Viral infections of the respiratory tract are a major cause of mortality in children in developing countries (Alemayehu et al., 2019) and a threat of occasional outbreak in immunocompromised patients and the elderly. Viruses causing these infections belong to the *Respirovirus* genus in the family *Paramyxoviridae*. In particular, human parainfluenza viruses type 1 (hPIV-1) causes moderate to severe respiratory tract illnesses (Karron and Collins, 2013) and is generally associated with laryngotracheobronchitis (also known as croup syndrome) (Schomacker et al., 2012; Abedi et al., 2015). Despite the ongoing efforts (Schmidt et al., 2011; Le Bayon et al., 2013), there is currently neither a vaccine nor a drug available to prevent or treat hPIV infections, respectively. Silencing the C gene in hPIV1 by reverse genetic technology has resulted in the attenuation of the virulence of the virus, demonstrating that the C protein is a primary factor in viral pathogenesis. Understanding the exact function of the C protein will thus contribute to not only the elucidation of viral pathogenesis but also the development of effective vaccines and antiviral agents.

Sendai virus (SeV), a pneumotropic virus of rodents and a murine counterpart of hPIV1, is the most extensively studied member of the *Respiroviru*s genus to determine the molecular and biological properties of hPIV1 and to develop an effective antiviral treatment against it (Lyn et al., 1991; Henrickson, 2003; Bajimaya et al., 2017). SeV also expresses the C proteins, which are translated from the P mRNA in a coding frame different from that of the P. SeVs express multiple C protein species, because their C open reading frames contain four independent translational start sites to produce a nested set of four carboxy-terminal proteins, namely C [amino acids (aa) 1 to 204], Y1 (aa 24 to 204), Y2 (aa 30 to 204), and C’(with a 11-aa addition to the N terminus of C), where C is the most abundant protein expressed in infected cells (Nagai et al., 2011; Lamb and Parks, 2013). The C proteins are categorized as non-essential accessory proteins but contribute greatly to virus replication *in vitro* and are indispensable for the *in vivo* multiplication and pathogenesis of the viral infection (Kurotani et al., 1998).

The C protein blocks type I IFN-stimulated JAK-STAT signaling pathway by inhibiting the activation of type I IFN receptor-associated kinases, JAK1 and TYK2, and the subsequent activation of STAT1 and STAT2 (Komatsu et al., 2000; Gotoh et al., 2003; Kitagawa et al., 2020). The C protein also regulates viral RNA synthesis and suppresses the production of IFN-inducing abnormal RNA species (double-stranded RNA (dsRNA), defective interfering RNAs, or both) (Komatsu et al., 2004; Takeuchi et al., 2008; Irie et al., 2010; Yoshida et al., 2015; Sanchez-Aparicio et al., 2018), possibly by interacting with the L protein, the viral RNA polymerase. This ability of the C protein to limit dsRNA also contributes to limited macrophage function, including the production of nitric oxide (NO), pro-inflammatory cytokines such as interleukin (IL)-6 and tumor necrosis factor (TNF)-α, and IFN-β, in infected macrophages (Odkhuu et al., 2018). Further research revealed that the *in vivo* depletion of airway macrophages during recombinant C gene-knockout SeV (SeVΔC) infection in mice resulted in the development of severe viral pneumonia (Sakuma et al., 2021). Therefore, the anti-macrophage activity of the C protein also appears to play an important role *in vivo*.

In the present study, we found another possible anti-macrophage activity. We observed that SeVΔC caused drastic morphological changes in infected RAW264.7 macrophages, similar to those induced via stimulation with Lipid A, a well-known activator of membrane ruffling and phagocytosis. In contrast, wild-type SeV (SeV WT) elicited minimal change compared to mock-infected cells. These findings indicated that the anti-macrophage activity of SeV C protein may be mediated through the restriction of phagocytosis, in addition to pro-inflammatory factor suppression. Thus, we sought to investigate the role of SeV C in macrophage morphological changes upon SeVΔC infection.

Herein, we aimed to examine the effect of SeV C protein on macrophage phagocytosis. The current results provide mechanistic insight for the development of therapeutics ameliorating disease severity during viral infection.

## Materials and Methods

### Cells and Viruses

ISGF3 reporter RAW-Lucia ISG cells (RAW264.7 murine macrophages) were used to monitor ISGF3 pathway activation. RAW-Lucia ISG-knockout (KO)-RIG-I cells and RAW-Lucia ISG-KO-MDA5 cells were used as RIG-I-deficient and MDA5-deficient RAW264.7 macrophages, respectively. These three cell lines were purchased from InvivoGen (San Diego, CA, United States) and cultured in Dulbecco’s modified Eagle’s medium (DMEM) containing 10% fetal bovine serum (FBS) and 200 μg/ml Zeocin, according to the manufacturer’s instruction. HEK293T cells and Vero cells were cultured in DMEM containing 10% FBS. Human glioblastoma cell line U118 cells were cultured in DMEM containing 10% FBS and 1 mM sodium pyruvate (Miyakoshi et al., 1990). SeV WT, a cDNA-derived Z strain, and mutant SeV 4C(−), in which all four C proteins have been knocked out (Kurotani et al., 1998), were propagated in Vero cells in the presence of 3 μg/ml trypsin. A new SeVΔC mutant strain, V35M, which does not generate dsRNA, was isolated from SeV 4C(-) stock in Vero cells via limiting dilution and characterized using U118 cells.

### Reagents and Immune Sera

Lipid A from *Salmonella enterica* serotype minnesota Re 595 was purchased from Sigma Chemicals (St. Louis, MO, United States). Mouse IFN-β and human IFN-β were purchased from PBL Assay Science (NJ, United States) and FUJIFILMWako (Osaka,Japan), respectively. Neutralizing antibodies against mouse IFN-β were purchased from PBL Assay Science. QUANTI-Luc for measuring ISGF3 (ISRE) promoter activity of RAW-Lucia ISG cells was purchased from InvivoGen. Polyethyleneimine (PEI) hydrochloride (MW 40,000) was purchased from Polysciences Inc. (Washington, PA, United States). GenomONE-GX was purchased from FUJIFILM Wako. The cells were transfected with 5′ triphosphate double stranded RNA (5′ppp-dsRNA), which is a synthetic ligand for the RIG-I (InvivoGen), using LyoVec, a cationic lipid-based transfection reagent (InvivoGen) according to the manufacturer’s instructions. Anti-SeV serum was prepared in rabbit by injecting a purified nucleocapsid suspension intravenously. Purified nucleocapsid suspension for immunization was prepared from SeV virion described previously (Compans and Choppin, 1967). Anti-C serum was used as previously prepared (Takeuchi et al., 2008).

### Phagocytosis Assay

Phagocytic activity in cells was determined using pHrodo (Red or Green) *Staphylococcus aureus* (*SA*) BioParticles (Thermo Fisher) according to the manufacturer’s instructions. The fluorescence of BioParticles increases dramatically under low pH conditions within mature phagolysosomes. Briefly, cells in a 48-well plate were infected with SeV strains at a multiplicity of infection (MOI) of 3, stimulated with Lipid A (200 ng/ml) or 5’ppp-dsRNA (10 or 20 ug/ml). After 24 h, cells were incubated with 80 μl of pHrodo-*SA* BioParticles (1 mg/ml) at 37°C for 1 h. The cells were then scraped, and fluorescence intensity was determined using FACS CantoII (Beckton Dickinson, NJ, United States). The mean fluorescent intensity (MFI) was analyzed using FlowJo software (FlowJo LLC, OR, United States).

### Cytotoxicity and Cell Proliferation Assays

The viral cytotoxicity and cell proliferation rates in infected cells were determined using the Viability/Cytotoxicity Multiple Assay Kit (Dojindo, Kumamoto, Japan) with culture medium and cells, respectively. For both controls, the same lysis buffer (containing TritonX-100) provided in the kit was used. The percentages of cytotoxicity and cell proliferation rates were determined according to the manufacturer’s instructions.

### Cell Staining

To observe the effect of the C protein on F-actin polymerization leading to membrane ruffling, cells in an 8-well chamber slide were infected with various SeV strains. At 24 h post-infection, the cells were fixed and permeabilized with PBS containing 4% paraformaldehyde (FUJIFILM Wako) for 10 min and 0.5% Triton X-100 for 5 min at 15-25°C. For observation of phagocytosis and F-actin polymerization, cells were fixed and permeabilized after incubation with BioParticles as described in the Phagocytosis assay section. The cells were then stained with Acti-stain 488 or 535 Fluorescent Phalloidine (Cytoskelton, CO, United States) and DAPI (Dojindo) according to the manufacturer’s instruction. For the detection of SeV antigens and endogenous RIG-I, the permeabilized cells were treated with anti-SeV serum and anti-RIG-I mouse monoclonal (D-12; Santa Cruz, TX, United States) antibody, respectively, followed by treatment with Alexa 647 Fluor-labeled anti-rabbit immunoglobulin G donkey serum (Abcam, LIFE Technology, CA, United States) and goat anti-mouse IgG-Cy5 (Invitrogen). The stained cells were mounted on slides with Fluoromount (Diagnostic Bio Systems, CA, United States) and then visualized under a confocal microscope LSM710 (Zeiss, Oberkochen, Germany). To observe the effect of SeV C protein on intracellular dsRNA generation, cells infected with various SeV strains were stained with a monoclonal antibody, J2, which specifically recognizes dsRNA of more than 40 bp (Schonborn et al., 1991), and visualized under a fluorescence microscope as previously described (Takeuchi et al., 2008).

### Immunoblot Analysis

Cells in a 24-well plate were infected with various SeV strains. At 24 h post-infection, cells were lysed in RIPA buffer (FUJIFILM Wako) and boiled with Laemmli sample buffer (Nakalai Tesque, Kyoto, Japan). Samples were resolved via SDS-polyacrylamide gel (7.5% or 10–20%) (FUJIFILM Wako) electrophoresis and then electroblotted onto a membrane (immobilon-P; Millipore, Bedford, MA). The membrane was blocked in Blocking One (Nakalai Tesque) for 30 min, followed by incubation at 15-25°C for 1 h with anti-RIG-I (D-12; Santa Cruz), anti-GFP (1E4; MBL, Aichi, Japan), and anti-actin (C4; Santa Cruz) mouse monoclonal antibodies, anti-C rabbit serum, and anti-SeV rabbit serum. The membrane was then incubated at 15–25°C for 1 h with a horseradish peroxidase-conjugated anti-mouse or anti-rabbit IgG antibody (GE Healthcare Life Sciences, Little Chalfont, United Kingdom). Immunoreactive bands were visualized using the enhanced chemiluminescence Western Lightning Ultra Substrate (Santa Cruz) and a FUSION SOLO S imaging system (VILBER LOURMAT, Collégien, France).

### Plasmids

The cDNA fragment encoding mouse RIG-I protein [1-926 aa] or mutant mouse RIG-I protein RIG-IC [231-926 aa] was used as previously described (Morita et al., 2020). The expression plasmid for RIG-I or RIG-IC with EGFP tag was created using the multicloning site of pCA7 with N-terminal EGFP tag by insertion of the cDNA fragment. The cDNA fragment for SeV Y^#^ [aa 35-204] was newly created by RT-PCR using specific primers. The cDNA fragment encoding SeV C [aa 1-204], Y1 [aa 24-204], Y2 [aa 30-204], Cm5 [aa 1-204], or Cm8 [1-204] protein was obtained from plasmids described previously (Kitagawa et al., 2020). The expression plasmid for C, Y1, Y2, Y^#^, Cm5, or Cm8 with EGFP tag was created using the multicloning site of pCA7 with N-terminal EGFP tag by insertion of the corresponding cDNA fragment. The pISREpuro3 plasmid carrying the puromycin-resistant gene was used as previously described (Kitagawa et al., 2018).

### Sequencing Analysis of the SeV V35M Strain

The sequence of the C gene in the V35M strain was determined via direct sequencing of the RT-PCR products from virion RNA using an RT-PCR kit with specific primers. The accession number is as follows: Entry ID LC654235 61619c1b3a01a5652dc15e5f.SeVZ_Cm.

### Statistical Analysis

Data are expressed as the mean ± standard deviation. Differences between two groups were analyzed using Student’s *t*-test, while those between three or more groups were evaluated via Tukey’s test or Dunnett’s test. Statistical significance was set at *P* < 0.05. All statistical analyses were performed using Microsoft Excel 2019 for Windows 10.

## Results

### SeV 4C(−) Activated Phagocytic Activity in Infected RAW264.7 Macrophages

**FIGURE 1:**
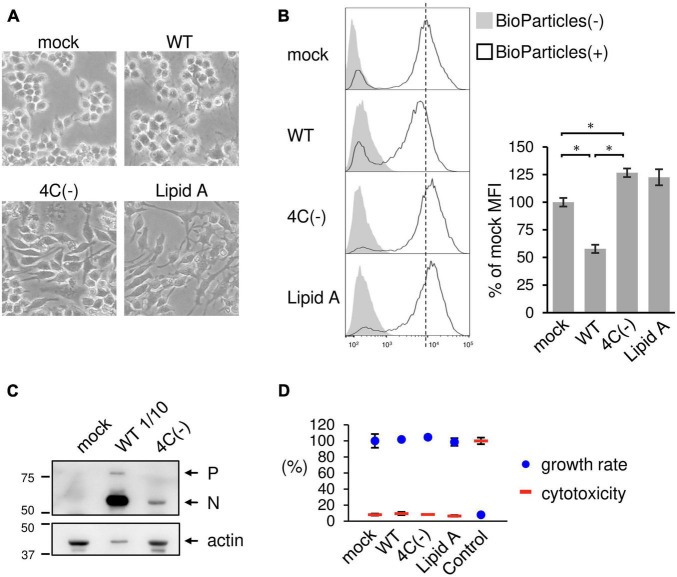
Activation of phagocytosis in SeV 4C(–)-infected RAW264.7 macrophages. **(A–D)** RAW-Lucia ISG cells were infected with the indicated SeV strains at MOI = 3 or treated with Lipid A (200 ng/ml) as a positive control for 24 h. **(A)** Morphological changes in cells were visualized under a light microscope. **(B)** The cells were then incubated with or without pHrodo-*SA* BioParticles. The phagocytic activity of cells was measured via flow cytometry (left panel). The results are expressed as a percentage of the MFI of mock-infected cells from three independent experiments (right panel). *P < 0.05, Tukey’s test. **(C)** Viral protein synthesis was determined via immunoblot analysis with anti-SeV serum. P and N indicate the SeV P and N protein, respectively. The SeV WT sample was subjected to SDS-PAGE at 1/10 of the volume of the SeV 4C(-) sample. **(D)** The cytotoxicity and cell proliferation rates were determined via LDH and NADH assays, respectively.

The recombinant C gene-knockout SeV (SeVΔC) 4C(−) strain, in which all four C protein genes had been knocked out, caused prominent morphological changes in RAW264.7 macrophages, including generally flattened shapes and two or more lamellipodial protrusions. In contrast, wild-type SeV (SeV WT) elicited minimal changes relative to mock-infected cells (Figure 1A). Stimulation with phagocytosis activator Lipid A resulted in the formation of lamellipodial protrusions as previously described (Kong et al., 2009; Mukherjee et al., 2009). This result suggested that the 4C(−) strain stimulated membrane ruffling, thereby leading to phagocytosis. In order to determine the effect of the SeV C protein on phagocytosis, RAW264.7 macrophages were infected with 4C(−) and then incubated with pHrodo-*Staphylococcus aureus* (*SA*) BioParticles as previously reported (Marvin et al., 2017). Infection with 4C(−) significantly increased the mean fluorescence intensity (MFI) as also observed in Lipid A-treated cells, while significantly lower fluorescence was observed in WT-infected cells compared to mock-infected cells (Figure 1B). To investigate the effect of viral replication on phagocytosis, viral protein synthesis was assessed via immunoblotting, as the knockout of all four C genes was previously reported to attenuate replication in other cell lines (Kurotani et al., 1998). Viral protein synthesis was considerably lower under 4C(−) infection relative to WT (Figure 1C). Thus, we investigated whether these differences in replication were associated with viral cytotoxicity and cell proliferation. There were no differences in cytotoxicity and proliferation rates between 4C(−) and WT infection in RAW264.7 macrophages (Figure 1D). Therefore, the disparity in phagocytosis did not appear to be due to differences in cytotoxicity and cell proliferation caused by replication, but rather due to other functions of the C protein.

### SeV 4C(−) Induced Actin-Rich Membrane Ruffle Formation Prior to BioParticle Exposure in RAW264.7 Macrophages

**FIGURE 2:**
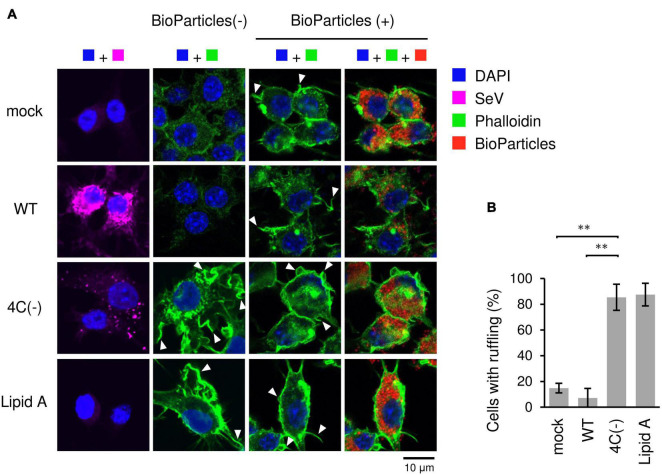
Actin-rich ruffle formation in SeV 4C(-)-infected RAW264.7 macrophages. **(A,B)** RAW-Lucia ISG cells were infected with the indicated SeV strains at MOI = 3 or treated with Lipid A (200 ng/ml) as a positive control for 24 h. **(A)** Cells incubated with pHrodo-*SA* BioParticles and stained for SeV antigen (purple), F-actin (phalloidin, green) and nuclei (DAPI, blue). Arrows indicate membrane ruffles. **(B)** The number of cells with membrane ruffles were counted among 40 cells in randomly selected fields, and the percentages of cells with membrane ruffles are shown. Data are presented as the mean ± SD of three independent experiments. ^**^P < 0.01, Tukey’s test.

The type of phagocytosis that occurs in response to invading pathogens requires dramatic changes in cell shape driven by actin polymerization and the subsequent formation of membrane ruffles. To examine whether the C protein affects membrane ruffle formation, we stained SeV-infected RAW264.7 macrophages with phalloidin before and after exposure to pHrodo-*SA* BioParticles. Phalloidin is used for staining actin filaments (also known as F-actin) which are formed by actin monomers (also known as G-actin). In mock- and WT-infected cells, membrane ruffling was induced only after BioParticle exposure (Figure 2). In contrast, 4C(−)-infected cells and Lipid A-stimulated cells exhibited ruffling even without BioParticle exposure. Further, membrane ruffling in 4C(−)-infected cells and Lipid A-stimulated cells was more prominent than that in WT- or mock-infected cells even after BioParticle exposure. Therefore, 4C(−) infection appeared to have a priming effect on macrophage phagocytosis prior to BioParticle exposure. Together, these results suggested that the C protein limits SeV-induced phagocytosis-associated membrane ruffling in macrophages.

### RIG-I Is Required for SeV 4C(−)-Activated Ruffling and Phagocytosis

**FIGURE 3:**
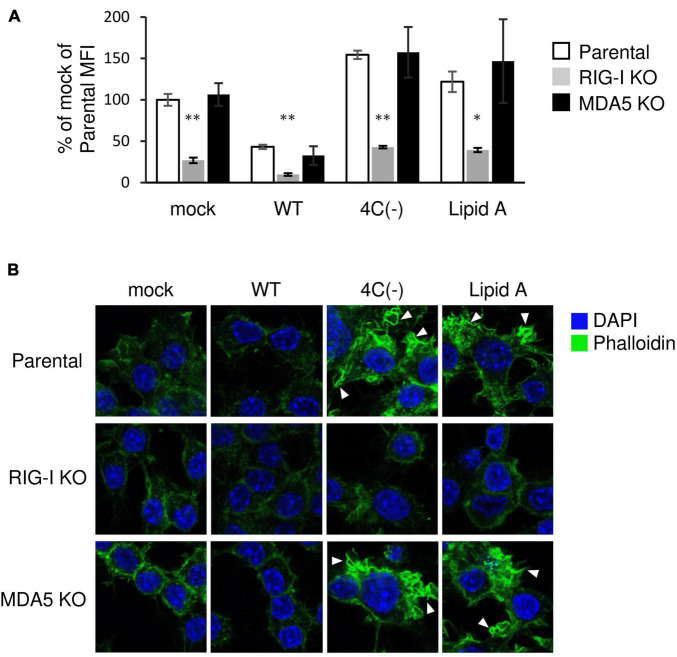
Role of RIG-I in the regulation of SeV 4C(–)-activated ruffle formation and phagocytosis. **(A,B)** Parent-, KO RIG- I-, or KO MDA5-RAW-Lucia ISG cells were infected with the indicated SeV strains at MOI = 3 or treated with Lipid A (200 ng/ml) for 24 h and incubated with or without pHrodo-*SA* BioParticles. **(A)** The phagocytic activity of cells was determined as described in Figure 1B. *P < 0.05, **P < 0.01 vs. Parental, Dunnett’s test. **(B)** The cells without BioParticles exposure were stained for F-actin (phalloidin, green) and nuclei (DAPI, blue).

RIG-I is essential for the Lipid A-stimulated phagocytosis of bacteria through facilitating actin polymerization and associated signaling (Kong et al., 2009). In order to investigate the role of RIG-I in 4C(−)-induced phagocytosis, we infected RAW-Lucia ISG-KO-RIG-I cells or RAW-Lucia ISG-KO-MDA5 cells with 4C(−), since the dsRNA generated by C gene-knockout parainfluenza virus type I activates RIG-I-like receptor (RLR) signaling (Boonyaratanakornkit et al., 2011; Sánchez-Aparicio et al., 2017). 4C(−)-activated phagocytosis was blocked in RIG-I knockout cells (Figure 3A), as previously reported for Lipid A-induced phagocytosis (Kong et al., 2009). This blockade was also observed under mock or WT infection. However, in RAW-Lucia ISG-KO-MDA5 cells, 4C(−)-activated phagocytosis was similar to that observed in the parental cell line. Similar results were obtained for membrane ruffle formation (Figure 3B). These results indicated that RIG-I, but not MDA5, is required for 4C(-)-induced ruffle formation and phagocytosis.

### SeV 4C(−) Induced Phagocytosis and Membrane Ruffling in an IFN-β-Independent Manner

**FIGURE 4:**
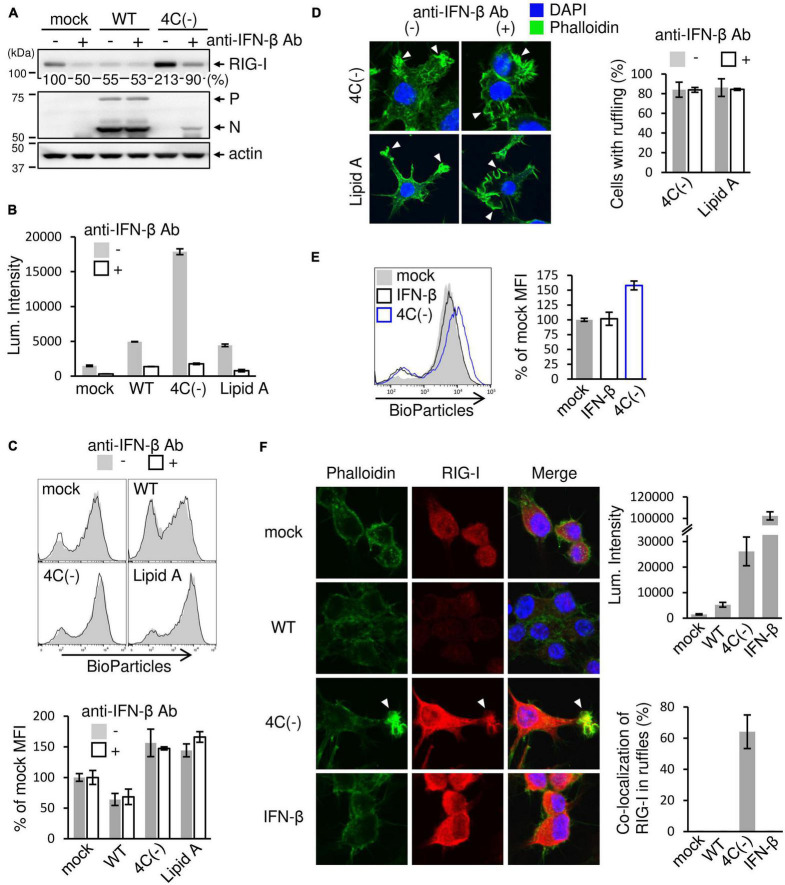
Effect of SeV-induced IFN-β on ruffle formation and phagocytosis. **(A-D)** RAW-Lucia ISG cells were infected with the indicated SeV strains at MOI = 3 or treated with Lipid A (200 ng/ml) as a positive control for 24 h. For anti-IFN-β antibody treatment, the culture medium contained neutralizing anti-IFN-β (2 × 10^3^ U/ml) antibodies throughout the experiments. **(A)** The cells were then lysed in lysis buffer, and lysates were subjected to immunoblotting with an anti-RIG-I antibody, anti-SeV serum, or an anti-β-actin antibody. The intensity band ratio of RIG-I to β-actin was estimated by a FUSION SOLO S imaging system. **(B)** ISGF3 (ISRE) promoter activity was determined by assessing the Luc activity in the culture media of cells. **(C)** The cells were then incubated with pHrodo-*SA* BioParticles and the phagocytic activity of cells was determined as described in Figure 1B. **(D)** The cells were stained for F-actin (phalloidin, green) and nuclei (DAPI, blue). The number of cells with membrane ruffles were determined as described in Figure 2B. *P* = ns anti-IFN-β antibody (+) vs. anti-IFN-β antibody (-), Student’s *t* test **(C,D)**. **(E,F)** RAW-Lucia ISG cells were infected with the indicated SeV strains at MOI = 3 or treated with IFN-β (500 IU/ml) for 24 h and incubated with or without pHrodo-*SA* BioParticles. **(E)** The phagocytic activity of cells was determined as described in Figure 1B. *P* = ns mock vs. IFN-β, Student’s *t*-test. **(F)** The cells without BioParticles exposure were stained for RIG-I (red), F-actin (phalloidin, green), and nuclei (DAPI, blue) (left panel). ISGF3 (ISRE) promoter activity was determined by assessing the Luc activity in the culture media of cells (right upper panel). The number of cells with RIG-I co-localized in membrane ruffles was determined as described in Figure 2B (right lower panel).

Results presented in the preceding sections suggested that the SeV C protein negatively regulates membrane ruffle formation, leading to phagocytosis through RIG-I signaling. Since 4C(−) induces a large amount of IFN-β in RAW264.7 macrophages (Odkhuu et al., 2018), IFN-stimulated gene product (ISG) RIG-I should also be upregulated. It was thus suggested that 4C(−)-induced IFN-β caused membrane ruffle formation, leading to phagocytosis. To assess this, we examined the effects of neutralizing antibodies against IFN-β on phagocytosis and ruffle formation. 4C(−) markedly upregulated RIG-I expression (Figure 4A). Antibody treatment clearly inhibited the increase in RIG-I expression, while also decreasing basal RIG-I expression in mock-infected cells, which indicated that the latter was upregulated via endogenous IFN-β. In contrast, WT infection downregulated basal RIG-I without antibody treatment, probably due to the inhibitory effect of C protein on JAK-STAT signaling. The blockade of SeV strain- or Lipid A-induced IFN-β through antibody treatment was confirmed based on Luc activity in infected RAW-Lucia ISG cells (Figure 4B). However, antibodies did not inhibit phagocytosis nor membrane ruffling in 4C(−)-infected and Lipid A-treated (Figures 4C,D). Conversely, the addition of an equivalent amount of exogenous IFN-β (500 IU/ml) did not elicit phagocytosis (Figure 4E). Since RIG-I associates with actin cytoskeleton and promotes membrane ruffling leading to phagocytosis, we compared the effects of the 4C(−) infection and exogenous IFN-β on endogenous RIG-I localization by confocal microscopic analysis. In 4C(−)-infected cells, endogenous RIG-I was upregulated and co-localized with F-actin in membrane ruffles in 4C(−)-infected cells, whereas it was upregulated but neither co-localization with F-actin nor membrane ruffles was observed in IFN-β-treated cells (Figure 4F). In contrast, the expression of RIG-I was decreased in WT-infected cells compared to uninfected cells, and neither co-localization with F-actin nor formation of membrane ruffles was observed in either cells. Taken together, these results suggested that the 4C(−)-induced RIG-I upregulation via IFN-β was not involved in phagocytosis and membrane ruffling. Given that RIG-I is required for both processes, 4C(−) might modulate its activation in an IFN-β-independent manner.

### SeV 4C(−) Promotes the Localization of RIG-I to Membrane Ruffles

**FIGURE 5:**
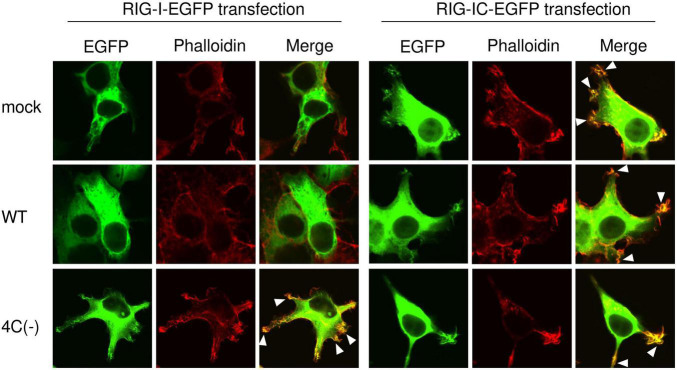
Localization of RIG-I to membrane ruffles in SeV 4C(–)-infected cells. HEK293T cells in an 8-well chamber slide were transfected with expression plasmids encoding EGFP-RIG-I (1-926 aa) or EGFP-RIG-IC (231-962 aa) [100 ng/well] using PEI. At 20 h post-transfection, cells were infected with the indicated SeV strains at MOI 3. At 20 h post-infection, cells were stained with phalloidin and visualized under a confocal microscope. EGFP-RIG-I and EGFP-RIG-IC (green) and F-actin (phalloidin, red). Data are representative of at least three independent experiments.

To confirm that IFN-responsive RIG-I upregulation is not required for membrane ruffling, we transiently expressed EGFP-RIG-I in HEK293T cells and examined the effect of SeV infection on membrane ruffling. Confocal microscopy revealed that EGFP-RIG-I markedly localized to membrane ruffles and clearly co-localized with F-actin in 4C(−)-infected cells, whereas no membrane ruffle association was observed in mock- or WT-infected cells despite sufficient RIG-I expression (Figure 5). When EGFP-RIG-IC was used, membrane ruffle formation and F-actin co-localization were observed not only in 4C(−)-infected, but also in mock- and WT-infected cells. RIG-IC is a C-terminal domain of RIG-I, which inhibits IFN-β production (Yoneyama et al., 2004) while constitutively activating membrane ruffle formation and phagocytosis (Kong et al., 2009). These results indicated that 4C(−) can induce membrane ruffling independently of RIG-I upregulation when the latter is above basal levels. Nevertheless, 4C(−) infection further activates RIG-I to enable membrane ruffle formation. Since the suppression of phagocytosis in WT cells correlated with RIG-I expression, basal or higher expression is required for phagocytosis.

### Role of dsRNA in Phagocytosis and Membrane Ruffling in RAW264.7 Macrophages Infected With 4C(−)

**FIGURE 6:**
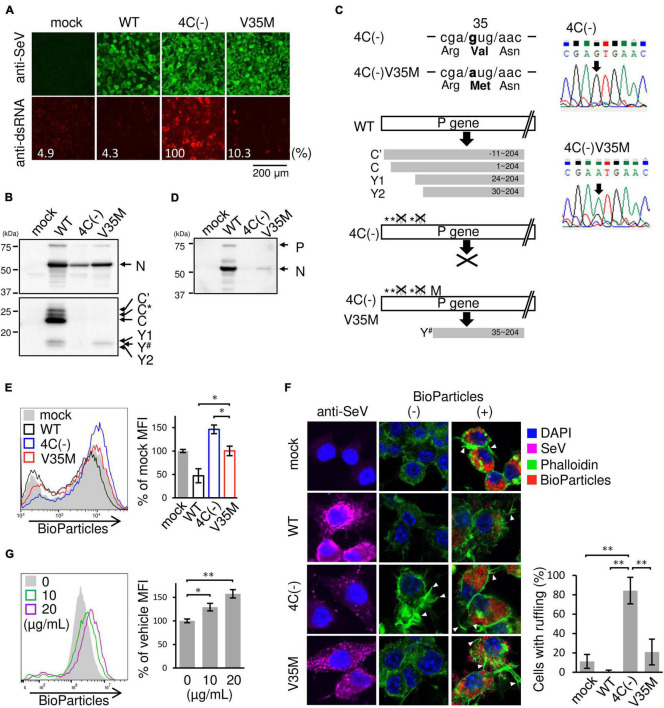
Role of dsRNA in phagocytosis by SeV-infected RAW264.7 macrophages. **(A,B,D–F)** U118 cells **(A,B)** or RAW-Lucia ISG cells (D-F) were infected with the indicated SeV strains at MOI = 3. **(A)** At 30 h post-infection, the cells were fixed and permeabilized. Intracellular dsRNA (red) or SeV antigens (green) were visualized via immunofluorescence staining with a mouse anti-dsRNA J2 monoclonal antibody or rabbit anti-SeV serum and analyzed under a fluorescence microscope. Fluorescence intensity was estimated by NIH images. **(B)** At 24 h post-infection, the levels of viral protein synthesis were determined via immunoblot analysis with anti-SeV or anti-C protein serum. Positions of C’, C, Y1, and Y2 bands were determined according to their estimated molecular weights. Y^#^ migrated faster than Y1 but slower than Y2. C* was determined as previously described (Takeuchi et al., 2008). **(C)** (left panel) The regions of C ORF containing the mutation in the 4C(–) and the cloned mutant strain V35M are shown. Schematic diagram of constructs of C proteins of the indicated SeV strains. (right panel) The electrographs of the region shown in (left upper panel) of the 4C(–) and V35M are shown. **(D)** At 24 h post-infection, the levels of viral protein synthesis were determined via immunoblot analysis with anti-SeV serum. **(E)** The cells were then incubated with pHrodo-*SA* BioParticles and the phagocytic activity of cells was determined as described in Figure 1B. *: *P* < 0.05, Tukey’s test. **(F)** The cells with or without BioParticles exposure were stained for SeV antigen (purple), F-actin (phalloidin, green) and nuclei (DAPI, blue). The number of cells with membrane ruffles was determined as described in Figure 2B. **(G)** RAW-Lucia ISG cells were transfected with 5-ppp-dsRNA. **P < 0.01, Turkey’s test. The cells were then incubated with pHrodo-*SA* BioParticles and the phagocytic activity was determined as described in Figure 1B. Data are representative of three independent experiments. *P < 0.05, **P < 0.01, Dunnett’s test.

SeV 4C(−) has been reported to produce dsRNA during viral transcription and replication, thereby inducing IFN-β, possibly via the RIG-I pathway (Takeuchi et al., 2008; Odkhuu et al., 2018; Morita et al., 2020). To investigate the role of dsRNA in membrane ruffle formation and phagocytosis, we attempted to isolate and characterize a new SeVΔC mutant from the 4C(−) strain that does not generate dsRNA. To this end, the 4C(−), which is normally passaged at 33°C in Vero cells, was passaged at 37°C, because we presumed that 4C(−) could be evolve at 37°C. A new SeVΔC mutant strain was isolated from the SeV 4C(−) stock in Vero cells via limiting dilution and was characterized using U118 cells. As shown in Figure 6A, low amounts of positive signals for dsRNA were observed in U118 cells infected with the new SeVΔC mutant strain, while clear positive signals for dsRNA were noted in 4C(−)-infected cells, as previously reported (Takeuchi et al., 2008). N protein expression in cells infected with the newly isolated SeVΔC mutant was approximately half that in cells infected with the WT, while expression in 4C(−)-infected cells was approximately one-fourth that of WT-infected U118 cells (Figure 6B). C′, C*, C, Y1, and Y2 proteins were not present in the new SeVΔC mutant as in 4C(−), but the Y^#^ protein was. Nucleotide sequence analysis of the isolated SeVΔC mutant C gene suggested that Val at the 35th position changed to Met, leading to the expression of Y^#^ (Figure 6C). Thus, the mutant was designated as the V35M strain. In IFN-β-producing RAW264.7 macrophages, unlike U118 cells, V35M strain viral protein expression was much lower than that of the WT, while much higher than that of 4C(−) (Figure 6D). To investigate the effect of the Y^#^ protein on phagocytosis, RAW264.7 macrophages were infected with V35M and then incubated with pHrodo-*SA* BioParticles, as shown in Figure 1B. The MFI of V35M-infected cells was not as high as that of 4C(−)-infected cells and was similar to that of mock-infected cells but did not decrease as much as that of WT-infected cells (Figure 6E). We stained V35M-infected cells with phalloidin before and after BioParticle exposure to investigate the effect of Y^#^ on membrane ruffling (Figure 6F). Membrane ruffling in V35M-infected cells was not induced without BioParticle exposure, as also observed for mock-infected cells and in contrast to 4C(−)-infected cells, which did exhibit ruffling. After BioParticle exposure, V35M-infected cells underwent prominent membrane ruffling, similar to that in mock-infected cells, which was less prominent than observed in 4C(−)-infected cells but more pronounced than in WT-infected cells. We next attempted to investigate whether 4C(−)-generated dsRNA could induce phagocytosis. However, as the amount of 4C-(−)-generated dsRNA is very low in infected cells, it is difficult to purify enough dsRNA for the transfection experiment. Therefore, 5′ppp-dsRNA, which is a synthetic ligand for RIG-I, was used. Transfection with 5′ppp-dsRNA increased phagocytosis in a dose-dependent manner (Figure 6G). Therefore, 4C(−)-generated dsRNA appears to have a priming effect on macrophage phagocytosis prior to BioParticle exposure via RIG-I. Further, these results indicated that the C protein is involved in the restriction of membrane ruffling and associated phagocytosis via limiting the generation of intracellular dsRNA.

### SeV C Protein Exerts Other Inhibitory Effects on Membrane Ruffling Besides Its Effect of Limiting dsRNA

**FIGURE 7:**
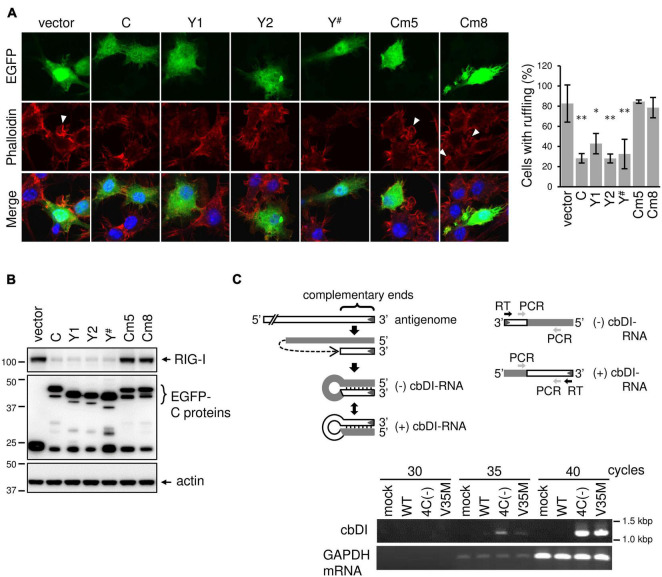
SeV C protein exerts other inhibitory effects on membrane ruffling besides its effect of limiting dsRNA. **(A)** RAW-Lucia ISG cells were transfected with the indicated plasmids using GenomONEGX. At 24 h post-transfection, the cells were treated with Lipid A for 24 h and then stained for F-actin (phalloidine, red) and with the indicated plasmids, together nuclei (DAPI, blue). *P < 0.05, **P < 0.01, vs. vector, Dunnett’s test. The number of EGFP-positive cells with membrane ruffles was determined as described in Figure 2B. **(B)** RAW-Lucia ISG cells were transfected with the indicated plasmids, together with pISREpuro3. At 24 h post-transfection, the cells were incubated in a medium containing puromycin (3 ug/ml) for 24 h. After removal of puromycin, surviving cells were treated with Lipid A (200 ng/ml) for 24h. The cell lysates were prepared and subjected to IB with an anti-RIG-I antibody or anti-EGFP antibody. **(C)** For the detection of cbDI RNA or mouse glyceraldehyde-3-phosphate dehydrogenase (mGAPDH) mRNA, total RNA was extracted from mock- or SeV-infected RAW264.7 macrophages using the High Pure RNA Isolation Kit (Roche). cDNA was then synthesized via reverse transcription of the total RNA with an RT primer (5′-ACAAGAGTTTAAGAGATATG-3′) or an oligo-dT primer. One tenth of the cDNA solution was subjected to PCR analysis of cbDI RNA and mouse GAPDH mRNA with the following primers: cbDI detection, a pair of cbDI-F (forward) (5′-TGATTAATAATGCTCGACAG-3′)and cbDI-R (reverse) (5′-CTTGTAAGTTTTTCTTACTA-3′). mGAPDH detection, a pair of mGAPDH_forward (5′-CATCACTGCCACCCAGAAGACCTG-3′) and mGAPDH_reverse (5′-ATGCCAGTGAGCTTCCCGTTCAG-3′). PCR was carried out at 98°C for 10 s, 50°C for 30 s, and 72°C for 30 s, for a total of 30 to 40 cycles.

Although V35M infection did not generate dsRNA, it did not inhibit phagocytosis as much as WT infection, suggesting a difference in function between the C proteins (C, Y1, Y2, and C′) and Y^#^. Since the results from phagocytosis inhibition by WT infection suggest that the C proteins exert other inhibitory effect(s) on phagocytosis, besides its effect of limiting dsRNA, we examined the effect of C, Y1, Y2, and Y^#^ expressions on membrane ruffling leading to phagocytosis. Because the transfection efficiency of RAW264.7 cells is very low, an EGFP-tagged C protein was introduced into RAW264.7 cells and membrane ruffling in response to Lipid A was assessed in EGFP-expressing cells. The membrane ruffling was inhibited in cells expressing the Y^#^ protein as well as in cells expressing the C, Y1, or Y2 protein (Figure 7A). We thus investigated the effects of the Y^#^ protein on RIG-I expression via immunoblotting (Figure 7B). The Lipid A-induced upregulation of RIG-I expression was clearly reduced by the expression of Y# as well as by the expression of the C, Y1, or Y2 protein. The inhibitory effects of C, Y1, Y2, and Y^#^ on membrane ruffling was in good agreement with the inhibitory effects of RIG-I expression, suggesting their ability to block JAK-STAT signaling may be involved. Therefore, we examined membrane ruffling using the C mutant (Cm) proteins Cm5 and Cm8, which have lost the ability to block JAK-STAT signaling (Kato et al., 2004; Kitagawa et al., 2020). In cells expressing the Cm5 or Cm8 protein, both membrane ruffling and RIG-I upregulation were not inhibited (Figures 7A,B). These results suggest that C, Y1, Y2, and Y^#^ inhibit membrane ruffling by blocking JAK-STAT signaling. However, the V35M strain did not inhibit phagocytosis as much as the WT strain, even though the Y^#^ protein possesses membrane ruffling-inhibitory activity like the C protein, suggesting that the V35M strain can activate phagocytosis independently of dsRNA. We previously reported that 4C(-) generates dsRNA (Takeuchi et al., 2008), while other research groups reported the generation of DI RNA, particularly copy-back (cb) DI RNA (Sánchez-Aparicio et al., 2017). Thus, we investigated the generation of cbDI RNA by estimating its levels in SeV-infected RAW264.7 macrophages via PCR with primers annealing to the cDNAs encoding cbDI(−) and cbDI(+) genomes, as described in the legend of Fig. 7C. The cbDI RNA levels were detected in 4C(−)- and V35M-infected cells, but not in WT-infected cells (Figure 7C). Further, cbDI RNA was not detected via immunofluorescence staining with the J2 monoclonal antibody specific for dsRNA (Figure 6A). These results indicate that 4C(-) generated both dsRNA and cbDI RNA, V35M generated only cbDI RNA, while WT virus did not generate either. Together, it could be inferred that although V35M activates phagocytosis through the generation of cbDI RNA, its activation may be counteracted by the inhibitory activity of Y^#^ protein, generating an illusion that it neither activates nor inhibits phagocytosis.

## Discussion

**FIGURE 8:**
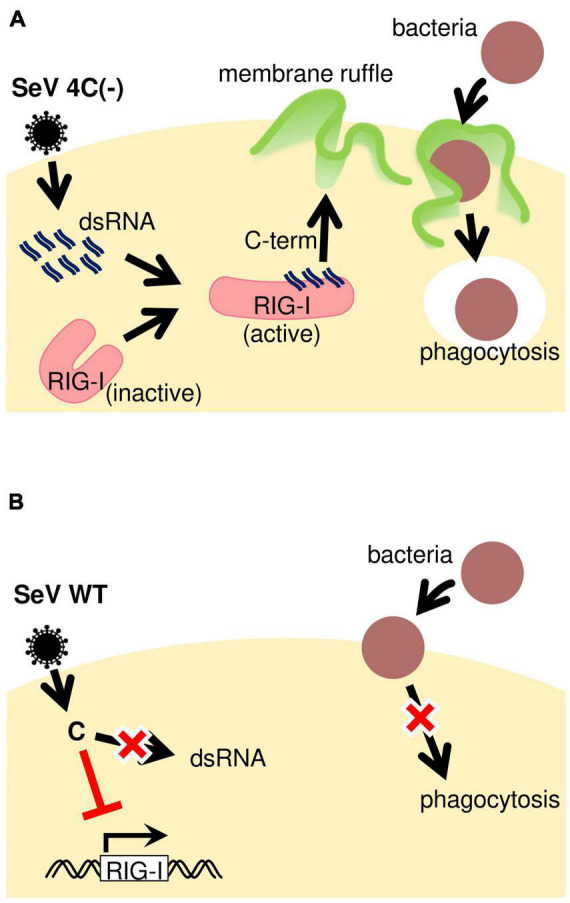
Schematic representation of phagocytosis regulation during SeV infection. **(A)** SeV4C(–) presumably activates RIG-I through dsRNA generated during infection, thereby inducing membrane ruffling. It is likely that 4C(–)-infected cells can efficiently phagocytose bacteria due to the priming effect dsRNA has on phagocytosis. **(B)** The SeV WT seems to suppress phagocytosis by blocking JAK-STAT signaling, besides not activating RIG-I in the first place due to no dsRNA generation during infection.

In the present study, we demonstrated that SeV 4C(−) infection of macrophages induced phagocytosis-associated membrane ruffle formation through the generation of intracellular dsRNA (Figure 8A). Our observations indicated that SeV C protein suppresses phagocytosis by limiting the generation of intracellular dsRNA. Together with our previous findings showing that the SeV C protein limits pro-inflammatory factor production through the inhibition of intracellular dsRNA generation (Odkhuu et al., 2018), the current results suggest that SeV C modulates both macrophage effector functions. Further, we demonstrated that host factors involved in the early clearance of SeVΔC in mice are not limited to IFN-β, but might also include macrophage-specific factors. Indeed, *in vivo* depletion of airway macrophages during SeV ΔC infection enhanced viral replication and infection pathogenesis in mice (Sakuma et al., 2021).

Our findings are similar to those of a previous study on IAV, which revealed that avirulent strains did not inhibit macrophage phagocytosis, unlike their virulent counterparts (Marvin et al., 2017). However, unlike avirulent IAV infection, wherein macrophage infection is abortive, avirulent SeV (SeVΔC) productively replicates in these cells (Odkhuu et al., 2014, 2018). Further, the antagonistic activity of SeV on phagocytosis depends not on whether its infection of macrophages is productive, but on whether it limits the generation of pathogen-associated molecular patterns during viral transcription and replication. Our current results indicated that the C protein affects phagocytosis. To our knowledge, this is the first study to demonstrate a relationship between SeV accessory protein C and phagocytosis.

SeV upregulated phagocytosis in the absence of the C protein, while suppressing it in the presence of the C protein (Figure 8). This cannot be explained only by the fact that the C protein completely limited dsRNA generation and is probably due to other effects on F-actin and membrane ruffling leading to phagocytosis. Ectopic expression of the indicated C proteins revealed that C, Y1, Y2, and Y^#^ inhibited membrane ruffling and RIG-I expression in response to Lipid A, but Cm5 and Cm8 did not, indicating that the ability of the C protein to block JAK-STAT signaling is important for other effects on membrane ruffling leading to phagocytosis. Unlike 4C(−), V35M expressed the Y^#^ protein (35-204 aa), which has an inhibitory activity on membrane ruffling. Therefore, V35M should inhibit phagocytosis, and the reason for the contradictory result might be the stimulatory effect of V35M-generated cbDI RNA. Since this cbDI RNA can activate phagocytosis only to a lesser extent than dsRNA plus cbDI RNA generated by 4C(−), this may offset the inhibitory effect and generate an illusion that V35M neither activates nor inhibits phagocytosis.

Alveolar macrophages are lung-resident immune cells that play important roles in host protection against respiratory virus infections. However, the underlying mechanisms through which alveolar macrophages modulate host inflammation, disease development, and tissue recovery are not well understood. Herein, we showed that the SeV C protein suppresses macrophage effector function. As the C protein is also encoded by other paramyxoviruses, such as human parainfluenza virus types 1 and 3, targeting it may represent a promising therapeutic strategy for the amelioration of viral infection-induced acute inflammation.

Nevertheless, the current study has limitations. First, we were unable to elucidate the mechanism of RIG-I-mediated phagocytosis activation via 4C(-)-generated dsRNA. Since 4C(−) activated phagocytosis in a manner similar to Lipid A treatment, we investigated Lipid A-associated signaling. Unfortunately, small GTPase Cdc42/Rac1, which is induced via Lipid A, could not be detected. While there are various types of phagocytosis, involving distinct small GTPases, there is limited information on the specific GTPase that contributes to membrane ruffling-driven phagocytosis (Mao and Finnemann, 2015). Thus, further research into the manner is necessary. Second, we could not determine whether the anti-macrophage activity of SeV C plays a crucial role in exacerbating disease. Our current and previous findings regarding the antagonistic effect of SeV C on phagocytosis and inflammatory factor production raise the critical question of whether these effects contribute to virulence and pathogenicity. To this end, we recently investigated the effects of macrophage depletion on SeVΔC pathogenesis and replication in mice. Depletion of airway macrophages via clodronate-loaded liposomes enhanced pathogenesis and viral replication *in vivo* (Sakuma et al., 2021). Therefore, the suppression macrophage function may represent a major strategy evolved by these viruses to enhance their pathogenicity.

In conclusion, the present study revealed that the SeV C protein limits phagocytosis by suppressing intracellular dsRNA generation. Together with our previous findings showing that the SeV C protein also limits pro-inflammatory factor production through the inhibition of intracellular dsRNA generation (Odkhuu et al., 2018), these results suggest that the anti-macrophage activity of SeV C plays an important role in virus replication and disease severity.

## Data Availability Statement

The datasets presented in this study can be found in online repositories. The names of the repository/repositories and accession number(s) can be found below: DDBJ LC654235/http://getentry.ddbj.nig.ac.jp/.

## Author Contributions

NM and TK designed the study and prepared and revised the manuscript. NM, YT, KT, and TK analyzed the data. NM, YT, KT, YK, RS, and NK performed the experiments.

## Conflict of Interest

The authors declare that the research was conducted in the absence of any commercial or financial relationships that could be construed as a potential conflict of interest.

## Publisher’s Note

All claims expressed in this article are solely those of the authors and do not necessarily represent those of their affiliated organizations, or those of the publisher, the editors and the reviewers. Any product that may be evaluated in this article, or claim that may be made by its manufacturer, is not guaranteed or endorsed by the publisher.
